# Marine Staurosporine Analogues: Activity and Target Identification in Triple-Negative Breast Cancer

**DOI:** 10.3390/md22100459

**Published:** 2024-10-05

**Authors:** Ru-Yi Chen, Li-Jian Ding, Yan-Jun Liu, Jin-Jin Shi, Jing Yu, Chang-Yun Li, Jian-Fei Lu, Guan-Jun Yang, Jiong Chen

**Affiliations:** 1 State Key Laboratory for Managing Biotic and Chemical Threats to the Quality and Safety of Agro-Products, School of Marine Sciences, Ningbo University, Ningbo 315211, China; 2311130002@nbu.edu.cn (R.-Y.C.); 2201130072@163.com (Y.-J.L.); 2311130039@nbu.edu.cn (J.-J.S.); 20102060117@lsu.edu.cn (J.Y.); 2211130023@nbu.edu.cn (C.-Y.L.); lujianfei@nbu.edu.cn (J.-F.L.); 2 School of Pharmacy, Health Science Center, Ningbo University, Ningbo 315211, China; dinglijian@nub.edu.cn

**Keywords:** staurosporine analogue, apoptosis, triple-negative breast cancer, cancer stem cells, cell migration, MAP3K11

## Abstract

Triple-negative breast cancer (TNBC) is a subtype of breast cancer with high mortality and drug resistance and no targeted drug available at present. Compound **4**, a staurosporine alkaloid derived from *Streptomyces* sp. NBU3142 in a marine sponge, exhibits potent anti-TNBC activity. This research investigated its impact on MDA-MB-231 cells and their drug-resistant variants. The findings highlighted that compound **4** inhibits breast cancer cell migration, induces apoptosis, arrests the cell cycle, and promotes cellular senescence in both regular and paclitaxel-resistant MDA-MB-231 cells. Additionally, this study identified mitogen-activated protein kinase kinase kinase 11 (MAP3K11) as a target of compound **4**, implicating its role in breast tumorigenesis by affecting cell proliferation, migration, and cell cycle progression.

## 1. Introduction

Breast cancer, the second leading cause of cancer-related deaths in women globally, exhibits considerable heterogeneity, ranging from stem cell-like to differentiated cells [1,2,3]. Approximately 10–20% of breast cancer cases are triple-negative breast cancer (TNBC), known for its aggressive behavior and lack of targeted therapies [4,5]. This subtype is responsible for a large proportion of deaths in breast cancer patients due to its high metastatic rate and poor prognosis [6,7]. Treatment typically involves surgery, radiotherapy, immunotherapy, or chemotherapy, but the resistance and poor outcomes associated with chemotherapy underscore the need for novel therapeutic targets and drug development [8].

The ocean possesses a unique environment and rich biodiversity not found in terrestrial habitats, with a diverse array of marine organisms serving as sources of natural products with distinct functions. Marine natural product chemistry has experienced significant growth in the past three decades [9]. Numerous natural products with novel structures derived from marine plants and animals have demonstrated a wide range of biological activities, thus driving the search for innovative natural products [10]. Various extracted ones from the marine sponge *Streptomyces fradiae* have been reported to exhibit anticancer activity. Fermented extracts of the marine bacterium *Streptomyces* sp. LY1209 displayed the most potent antiproliferative effects on Molt-4 leukemia cells [11]. Additionally, natural indolocarbazole analogues obtained from marine sediment strains of *S. fradiae* were found to inhibit pancreatic cancer (PC) tumor growth through selective inhibition of PKCθ/δ, a known tumor promoter, with its increased expression serving as a negative prognostic indicator for human breast cancer; thus, it is speculated that this compound may also inhibit breast cancer growth [12]. Compounds **1** and **2** exhibited significant cytotoxicity against the estrogen receptor-positive breast cancer cell line MCF-7, while compound **2** also demonstrated strong inhibitory effects on Adriamycin-resistant MCF-7 (MCF-7/ADR) cells [13]. But their mechanism of action for ER-negative breast cancer cell lines is unclear in terms of chemotherapy resistance.

In the present study, we isolated two additional compounds from *Streptomyces* sp. NBU3142 in a marine sponge, and we evaluated the cytotoxicity of these four compound libraries on chemoresistant and chemosensitive TNBC cells by CCK8. Compound **4** was found to possess better cytotoxicity against drug-resistant and non-drug-resistant breast cancer cell lines. Its mechanism of action is to inhibit breast cancer cell migration, reduce cell stemness, induce apoptosis, and stimulate cell cycle arrest by targeting MLK3, and the results of studies suggest that compound **4** is a potential therapeutic agent for triple-negative breast cancer.

## 2. Results

### 2.1. Structure of Compound ***4***

Compounds **1** and **2** have demonstrated significant inhibitory effects on ER-positive breast cancer cells [13]. The Log P of compounds **1**–**4** are the following: Compound **1**: 3.79 > Compound **2**: 3.07 > Compound **4**: 2.78 > Compound **3**: 1.98 (https://www.molecular-modelling.ch/, accessed on 14 September 2024). Compounds **1**,**2** have a methyl group, and therefore compounds **1**,**2** are more hydrophobic than compound **4**, suggesting poorer water solubility resulting in poorer absorption. The hydrophobicity of compounds **1**,**2** is higher than that of compound **4**, indicating that **1**,**2** is less water-soluble, resulting in poorer absorption. From the binding energy of molecular docking (lower binding energy), it is possible that the presence of the methyl group of compounds **1**,**2** causes spatial site resistance in the binding of the compounds to protein. Compound **3** has two more hydroxyl groups than compound **4**, and therefore compound **3** is less hydrophobic than compound **4**, which is not favorable for the binding of small molecules to proteins; therefore, compound **4** has a better spatial structure.

### 2.2. Compound ***4*** Exhibited the Best Inhibitory Activity against TNBC Cell Lines

**Figure 1:**
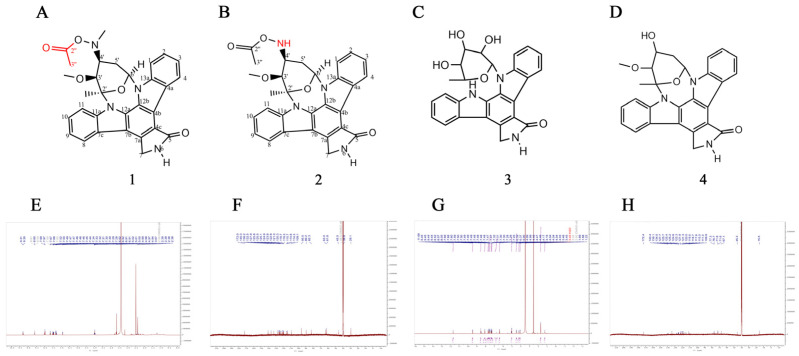
Structures of compounds **1**–**4**. (**A**–**D**) The chemical structures of compounds **1**–**4**, (**E**) ^1^H NMR spectrum of compound **4**, (**F**) ^13^C NMR spectrum of compound **4**, (**G**) ^1^H NMR spectrum of compound **3**, (**H**) ^13^C NMR spectrum of compound **3**.

**Figure 2:**
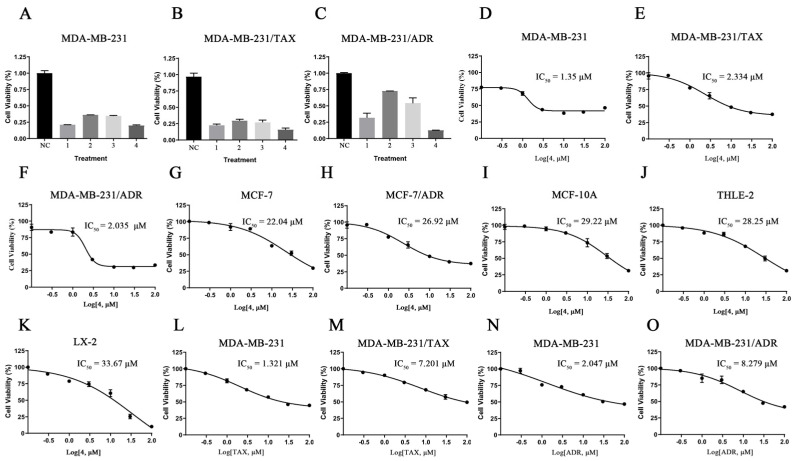
The effects of compound **4** on cytotoxicity against several breast cancer cell lines. (**A**–**C**) The cytotoxic effects of the four compounds against MDA-MB-231 and two drug-resistant MDA-MB-231 cells at a concentration of 10 μM. (**D**–**H**) The half-inhibitory concentration of compound **4** against different breast cancer cells. (**I**–**K**) The half-inhibitory concentration of compound **4** against non-cancer cells. (**L**,**M**) The half-inhibitory concentration of paclitaxel against MDA-MB-231 and paclitaxel-resistant 231 cells. (**N**,**O**) The half-inhibitory concentration of Adriamycin against MDA-MB-231 and Adriamycin-resistant 231 cells.

To investigate the anticancer activity of these four compounds (Figure 1A–D) on TNBC cells, we conducted a CCK8 assay. The results indicated that compound **4** exhibited the most potent inhibitory effect on the three TNBC cell lines at a concentration of 10 μM (Figure 2A–C). Consequently, we selected compound **4** for further toxicity validation against other breast cancer cell lines. Treatment with different concentrations of compound **4** (0, 0.1, 0.3, 1, 3, 10, 30, 100) for 48 h revealed that compound **4** inhibited the cell viability of several breast cancer cell lines (MDB-MB-231, MDB-MB-231/ADR, MDB-MB-231/TAX, MCF-7, MCF-7/ADR) in a dose-dependent manner (Figure 2D–H). However, in comparison, compound **4** showed a better toxic effect on triple-negative breast cancer cells (compound **4** (IC_50_: 1.35) was found to be more toxic to MDA-MB-231 cells than staurosporine (IC_50_: 7.67 μM)). The lower toxic effect of compound **4** on normal mammary epithelial cells as well as on normal hepatocytes of both human species can be seen in Figure 2I–K (Figure 2L–O shows a resistant effect of two resistant cell lines of MDA-MB-231 cells). Therefore, we selected 231 as well as paclitaxel-resistant 231 as the experimental subjects in this paper.

### 2.3. The Effects of Compound ***4*** on the Apoptosis of TNBC Cell Lines

**Figure 3:**
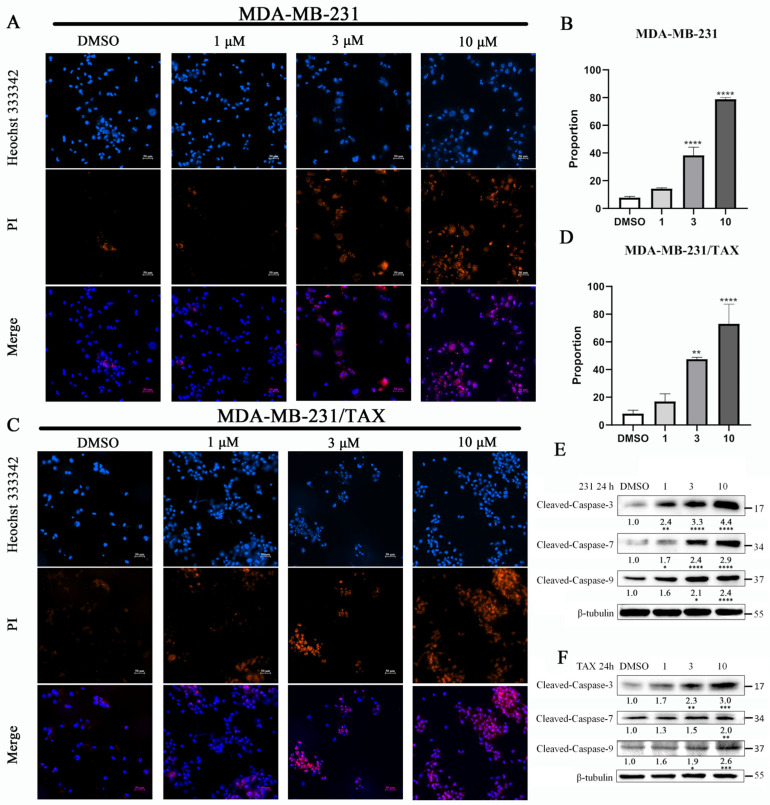
The effects of compound **4** on the apoptosis of TNBC cell lines. (**A**,**C**) The effects of compound **4** on the apoptosis of MDA-MB-231 and MDA-MB-231/TAX cells detected by Heochst33342/PI-stained assay after 24 h treatment. (**B**,**D**) The proportion of apoptosis (red to blue) cells in (**A**,**C**). (**E**) The apoptotic protein levels in MDA-MB-231 and (**F**) MDA-MB-231/TAX cells were analyzed by Western blotting after 24 h treatment with compound **4**. Data are expressed as the mean ± SD. * *p* < 0.05, ** *p* < 0.01, *** *p* < 0.001 **** *p* < 0.0001 (Student’s *t*-test). Scale bar: 50 μm.

These caspases are known to play a crucial role in the apoptotic process. The inhibitory effect of compound **4** on MDA-MB-231 cells as well as drug-resistant MDA-MB-231 cells was analyzed using immunofluorescence and Western blotting. Cells treated with varying concentrations of compound **4** were stained using Hoechst33342/PI to assess apoptosis. The results indicated an increase in the number of PI-stained cells and a corresponding rise in the proportion of apoptotic cells with higher concentrations of the drug (Figure 3A–D). This finding was further corroborated by immunoblotting for apoptotic markers, specifically caspase-3, caspase-7, and caspase-9, in both MDA-MB-231 cells and their drug counterparts [14]. The results found compound **4** dramatically elevated the levels of cleaved caspase-3, -7, and -9 proteins in a dose-dependent manner compared to control group (Figure 3E,F). Notably, the activities of these caspases were positively correlated, consistent with previous studies [15,16]. Based on these results, we conclude that compound **4** induces apoptosis by enhancing the activity of caspase-9, -7, and -3 in MDA-MB-231 and MDA-MB-231/Tax cells.

### 2.4. The Effects of Compound ***4*** on the Cell Cycle of TNBC Cells

**Figure 4:**
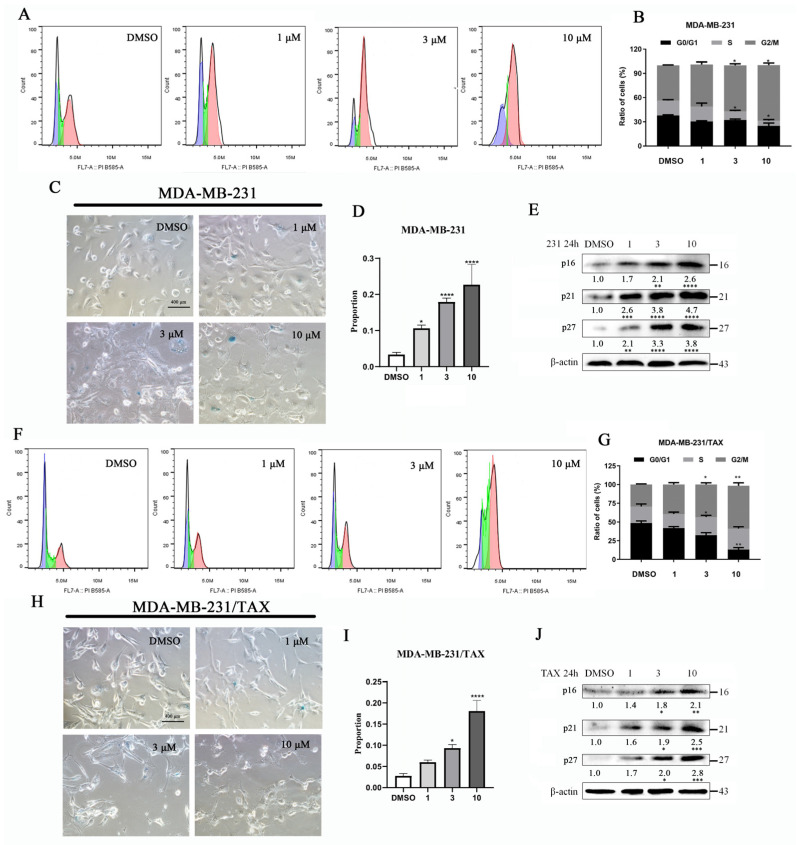
Compound **4** increases G2/M cell population and induces cellular senescence in TNBC cells. MDA-MB-231 cells were treated with compound **4** and drug-resistant MDA-MB-231 cells showed an increased rate of entering the G1 phase of the cell cycle (**A**,**B**,**F**,**G**) and senescent cells (**C**,**D**,**H**,**I**) as compared to the DMSO control. Protein levels of p16, p21, and p27 were measured by Western blotting analysis in MDA-MB-231 cells treated with compound **4** (**E**) and in drug-resistant MDA-MB-231/TAX cells (**J**). Data are expressed as the mean ± SD. * *p* < 0.05, ** *p* < 0.01, *** *p* < 0.001 **** *p* < 0.0001 (Student *t*-test). Scale bar: 400 μm.

The anti-tumor effects of staurosporine analogues are believed to involve the regulation of cell cycle progression [17]. To investigate whether structurally similar compounds inhibited cell growth and induced cancer cell senescence through cell cycle blockade, we assessed cell cycle changes and analyzed the expression of cell cycle-related proteins using Western blotting. Our findings revealed that the levels of p16, p21, and p27 proteins increased in both MDA-MB-231 and drug-resistant MDA-MB-231/TAX cells as the concentration of compound **4** treatment increased, with peak levels observed at a concentration of 10 μM (Figure 4E,J). Following treatment with varying concentrations of compound **4**, we analyzed the distribution of DNA content in the cells at different time points using flow cytometry. The results indicated that compound **4** treatment significantly increased the proportion of cells in the G2/M phase, with 66.2% and 51.3% of cells exhibiting 4N DNA content at the 10 μM concentration, respectively (Figure 4A,B,F,G). Concurrently, the population of cells in the G1/S phase was significantly reduced, and the ratio of senescent cells increased with higher concentrations of the drug treatment (Figure 4C,D,H,I). These results suggest that compound **4** induces G2/M phase arrest and promotes cellular senescence in both MDA-MB-231 cells and MDA-MB-231/TAX cells by transcriptionally regulating the levels of p16, p21, and p27 in a dose-dependent manner.

### 2.5. The Effects of Compound ***4*** on the Stemness of TNBC Cells

**Figure 5:**
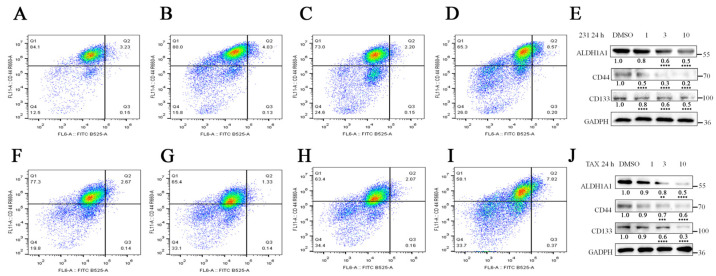
The effect of compound **4** treatment on the stemness of TNBC cells. (**A**–**D**) Flow cytometry results of CD44 and CD24 staining of MDA-MB-231 cells treated with different concentrations of compound **4**. (**F**–**I**) Flow cytometry results of CD44 and CD24 staining of paclitaxel-resistant MDA-MB-231 cells treated with different concentrations of compound **4**. Changes in protein content of stem cell indicators ALDH1A1, CD44, and CD133 in (**E**) MDA-MB-231 and (**J**) MDA-MB-231/TAX treated with different concentrations of compound **4** (1 μM, 3 μM, 10 μM) compared to the control. Data are expressed as the mean ± SD. ** *p* < 0.01, *** *p* < 0.001 **** *p* < 0.0001 (Student’s *t*-test).

Cancer stem cell-like cells (CSCs) represent a small subpopulation within various cancers, including breast cancer, characterized by their high tumorigenicity and stem cell properties such as their self-renewal and ability to form tumor spheroids [18]. CSCs are identified using specific cell surface markers or membrane transporter proteins, with cells expressing CD44^high^ and CD24^low^ on their surface suggested as being breast CSCs [19,20]. To evaluate the effect of compound **4** on CSCs, we quantified the difference in CD44^+^/CD24^–^ cell ratios between the MDA-MB-231 cells and MDA-MB-231/TAX cells treated with compound **4** for 24 h using flow cytometry. The results showed that compound **4** significantly reduced the ratio of CSCs in both TNBC cell lines (Figure 5A–D,F–I). Additionally, we analyzed the changes in surface CSC marker proteins (ALDH1A1, CD133, CD44) using Western blotting after compound **4** treatment. The results elucidated that theses CSC biomarkers showed a decreasing trend with the increase in drug concentration (Figure 5E,J).

### 2.6. The Effects of Compound ***4*** on the Cell Migration of TNBC Cells

**Figure 6:**
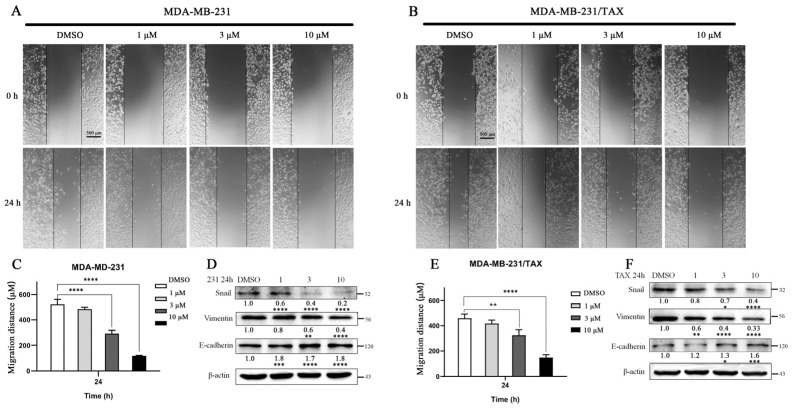
The effect of compound **4** on the migratory ability of TNBC cells. The migratory distances of MDA-MB-231 and MDA-MB-231/TAX cells were detected after 24 h treatment with compound **4** at concentrations of 1 μM, 3 μM, and 10 μM (**A**–**C**,**E**). The protein content of markers of cell migration was detected by Western blotting (**D**,**F**). Data are expressed as the mean ± SD. * *p* < 0.05, ** *p* < 0.01, *** *p* < 0.001 **** *p* < 0.0001 (Student’s *t*-test). Scale bar: 500 μm.

A wound-healing assay was conducted to evaluate the effect of compound **4** on the TNBC cell lines. The results indicated a significant decrease in the migration rate of both MDA-MB-231 and MDA-MB-231/TAX cells following compound **4** treatment compared to DMSO-treated groups, with a negative correlation observed with drug concentration (Figure 6A–C,E). The Western blotting results revealed that the protein levels of migration markers Snail and Vimentin decreased, while E-cadherin levels increased with higher concentrations of compound **4** administration. In summary, compound **4** inhibits cell migration by downregulating Snail and Vimentin and upregulating E-cadherin (Figure 6D,F).

### 2.7. Target Identification and Binding Mode Analysis of Compound ***4***

**Figure 7:**
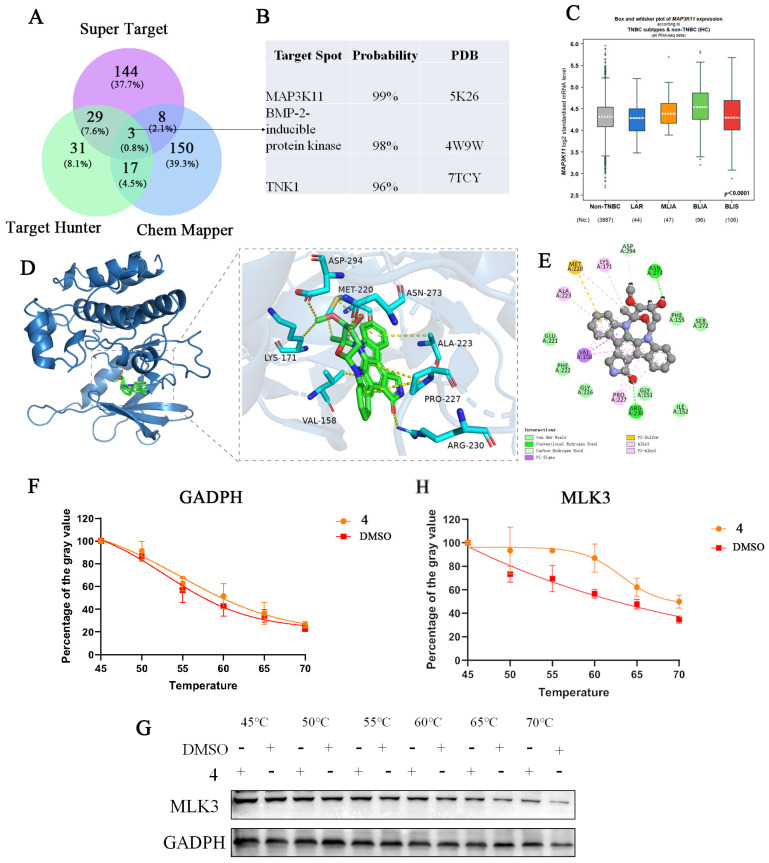
The target prediction of compound **4** and its binding mode to target were analyzed. (**A**) The potential targets of compound **4** predicted by the software. (**B**) The information of the candidate targets. (**C**) The RNA expression changes in the target MLK3 in TNBC and non-TNBC cell lines. * *p* < 0.0001. (**D**) The simulated binding sites of compound **4** to MLK3. (**E**) The potential binding sites of compound **4** to MLK3. (**F**) GADPH and (**H**) MLK3 cell thermal shift assays on the binding capacity of compound **4**. (**G**) Western blotting pictures of compound **4** treated at different temperatures and MLK3.

Target Hunter is a target finder based on the 2D structural similarity of small molecules, while Chem Mapper is a target prediction tool that utilizes the SHApe-FeaTure Similarity (SHAFTS) method, focusing on predicting multi-pharmacological effects and modes of action of small molecules by translating the shapes of three-dimensional molecules into comparable data. Super Target integrates DrugBank, BindingDB, and SuperCyp, creating a database that collects information about drug–target relationships. By integrating the targets predicted by these three databases, we identified three genes: mitogen-activated protein kinase kinase kinase 11 (MAP3K11, also named MLK3), non-receptor tyrosine protein kinase TNK1, and BMP-2-inducible protein kinase (Figure 7A,B). The search and analysis of information regarding these genes, along with probability analysis of the targets predicted by the software, revealed that MAP3K11 was the most likely target. We then analyzed the RNA expression levels of MAP3K11 in TNBCs and non-TNBCs (non-TNBC). The “Box and Whisker” display in the bc-GenExMiner v5.1 database indicated that MAP3K11 was expressed in both TNBC (IHC) and non-TNBC (IHC) (Figure 7C).

To explore the interaction of compound **4** with MLK3 protein targets, molecular modeling was performed using MOE 2019 software. The binding results indicated that the small-molecule compounds could enter the binding domains of the MLK3 target proteins. Specifically, residues Asn273 and Arg230 on the MLK3 protein receptor formed hydrogen bond interactions with the compounds, while residues Asp294 and Asn273 formed hydrocarbon interactions. Additionally, residues Lys171, Ala223, Pro227, Val158, and Met220 on the receptor formed hydrophobic interactions with compound glycosides. Residue Val158 also formed Pi–Sigma interactions, and residue Met220 formed electrostatic interactions with the compound. The molecular docking energy derived from these binding modes was −8.0919 kcal/mol (Compound **1**: −3.842 > Compound **2**: −4.313 > Compound **3**: −6.66 > Compound **4**: −8.0919), indicating strong binding activity between the small-molecule active ingredient and the MLK3 protein target (Figure 7D,E). CETSA is based on the thermal stability of the protein target through compound binding [21]. It was found that the protein from the compound **4**-treated group was more stable after heating compared with the control group by the CETSA test, which verified that compound **4** could inhibit cell proliferation through MLK3 (Figure 7F,G).

## 3. Discussion

TNBC is an invasive subtype of breast cancer notoriously difficult to treat due to its high rates of metastasis and recurrence, resulting in the worst prognosis among all breast cancer subtypes [22]. Despite the availability of several effective treatment options, including surgery, radiotherapy, chemotherapy, and endocrine therapy, the associated side effects are significant, and the mortality rate for breast cancer patients remains high [21]. Therefore, there is an urgent need for new drugs and treatment strategies.

Marine natural products are reported to exhibit superior biological properties compared to their terrestrial counterparts [23]. Among these, indolocarbazole-type alkaloids, which arise from the oxidative dimerization of tryptophan, represent a significant class of structurally intriguing and biologically active natural products. These compounds have been reviewed for their pharmacological activities, including their antidiabetic [24], antimalarial potential [25], antidepressant, anxiolytic [26], and immunomodulatory activity [27]. Notably, several indolocarbazole derivatives demonstrate potent anticancer activity against tumors and drug-resistant cancer cell lines [28,29]. Since the identification of the first member of this class, staurosporine, over one hundred natural indolocarbazole products have been discovered from various marine sources, including sponges, periphyton, fungi, cyanobacteria, and algae. The phylum Porifera, which comprises approximately 8500 marine sponge species, is particularly notable for producing a diverse array of secondary metabolites with remarkable anticancer, antibacterial, antifungal, antioxidant, and other therapeutic activities [30,31]. Several indolocarbazole derivatives have progressed to phase II/III clinical trials or have been approved as clinically effective drugs for various cancers, including being potent inhibitors of protein kinase and topoisomerase-1, such as rebeccamycin and staurosporine [31]. The compound studied in this paper, referred to as compound **4**, is one of these derivatives, extracted from the marine sponge *Streptomyces* spp. The role of this compound was elucidated through investigations of apoptotic phenotypes, stem cell characteristics, cell cycle blockade effects, and the migratory capabilities of cancer cells. Staurosporine, an indolocarbazole compound, has been previously studied in breast cancer cell lines, including HBL-100 [32], MCF-7 [33], and T47D [34], and is also present in TNBCs [29]. Therefore, our primary focus was on the effects of compound **4** on TNBC cells, specifically MDA-MB-231. The standard clinical treatment for TNBC typically involves a combination of paclitaxel and anthracyclines. Thus, it was crucial to investigate the efficacy of compound **4** on paclitaxel-resistant cells. In this study, we concentrated on paclitaxel-resistant MDA-MB-231 cells to assess the inhibitory effects of compound **4** on both normal TNBC cells and their drug-resistant counterparts.

Apoptosis is a genetically controlled form of programmed cell death that involves the activation, expression, and regulation of a range of genes [35]. The enzymes that execute apoptotic instructions are cysteine proteases, which are cleaved from inactive pro-cysteine proteins either autocatalytically or by other cysteine proteases to produce the active enzyme [36]. Caspase-3, activated by various pro-apoptotic stimuli, plays a crucial role in the execution phase of apoptosis [37,38]. It binds to apoptotic protease-activating factor 1 and pro-caspase-9 to form an “apoptosome” [39]. This complex formation stimulates the oligomerization of pro-caspase-9 and its autocatalytic activation, promoting the activation of downstream caspases (e.g., caspase-3 and -7) [40]. Thus, caspase-3, -7, and -9 can serve as biochemical markers of the execution phase of apoptosis. Indolocarbazole alkaloids, including compound **4**, exhibited cytotoxic activity against MDA-MB-231 breast cancer cell lines. To elucidate the mechanism of cytotoxicity, Hoechst 33342/PI staining was performed to assess apoptosis, further confirmed by immunoblotting for the apoptotic markers caspase-3, -7, and -9. Western blotting analysis revealed the processing of pro-caspase-9 into its active form, with the downstream activation of effector caspase-3 and -7, and a significant increase in the protein levels of cleaved caspase-3, -7, and -9. These results indicated that compound **4** effectively induced apoptosis in MDA-MB-231 cells and exhibited a pro-apoptotic effect on drug-resistant MDA-MB-231 cells. Treatment-induced senescence is classified as premature senescence, and Milanovic et al. suggested that the induction of premature senescence in tumor cells not only implies growth arrest in these cells, but may be associated with disease stabilization [41]. In some cases, senescent cells can escape growth arrest and re-enter the cell cycle [42]. Experiments have shown that post-senescent cancer cells have a higher potency for tumorigenesis than never-senescent cancer cells, as a result of the acquisition of stemness-related properties in a cell-autonomous manner during senescence-associated reprogramming [43]. Meanwhile, our results showed that compound **4** treatment not only significantly reduced the stemness and migratory ability of TNBC cells, but also markedly altered the senescent morphology of TNBC cells by β-galactosidase assay, suggesting that the cellular senescence induced by compound **4** treatment was irreversible. Cellular senescence is an important tumor suppressor process that prevents damaged cells from undergoing aberrant proliferation [44]. p16 and p21 levels are always elevated in senescent cells and are two key regulators of senescence [44]. p27 is a potent inhibitor of several cyclin–CDK complexes [45]. Other markers of senescence include a large flat morphology and the induction of senescence-associated β-galactosidase activity [46], and the antiproliferative activity of indolocarbazole-like staurosporine is due to its significant effect on the cell cycle by interfering with multiple components of the CDK system. This interference leads to cell arrest at G2/M or G0/G1 in a cell type-dependent manner [47,48]. Compound **4** primarily induced a delay in G2/M phase progression in MDA-MB-231 cells. Flow cytometry with PI staining and protein immunoblotting of cell cycle markers were employed to observe cell cycle changes. The flow cytometry results indicated a significant increase in the proportion of MDA-MB-231 and drug-resistant MDA-MB-231 cells in the G2/M phase, suggesting that compound **4** induced a blockade of cell entry into mitosis and accumulation in the G2/M phase. Western blotting revealed a significant increase in the protein levels of p16, p21, and p27, with increasing concentrations of compound **4**, indicating that treatment with compound **4** significantly hindered cell cycle progression. Furthermore, we verified the senescence of drug-treated and drug-resistant MDA-MB-231 cells by detecting changes in β-galactosidase activity, finding that increased drug concentration deepened cellular senescence (cells lost their fusiform shape and became irregular, with a huge increase in their size, showing an irreversible trend). These results illustrate that compound **4** can promote cellular senescence through G2/M phase blockade, with similar effects observed in drug-resistant cells.

The earliest CSCs isolated and characterized in solid tumors were from breast cancer. These breast cancer stem cells (BCSCs) are characterized by the cell surface markers CD44^high^, CD24^low^, CD133^high^, and aldehyde dehydrogenase (ALDH) enzymatic activity [49,50]. CD44 is a type I transmembrane glycoprotein that regulates cell adhesion and interactions between cells and the extracellular matrix. BCSCs expressing CD44^+^/CD24^–^ have a greater capacity for tumor development in mice, prompting us to examine their expression status by flow cytometry [51]. LDH, a detoxifying enzyme responsible for intracellular aldehyde oxidation, plays a role in the early differentiation of stem cells by oxidizing retinol to retinoic acid [52,53]. Both ALDH1A1 and CD133 have been suggested as potential markers for malignant breast stem cells [50,54]. Compound **4** treatment significantly reduced the proportion of CD44^+^/CD24^–^ cells and protein content of stem cell markers CD44, CD133, and ALDH1A1, suggesting that compound **4** could reduce the population of cancerous breast cells by inhibiting both MDA-MB-231 and drug-resistant MDA-MB-231 cells.

E-cadherin plays an important role in maintaining normal epithelial cell adhesion as well as tissues’ structural integrity [55], and it also exhibits an inhibitory effect on tumor metastasis and is regarded as a tumor metastasis suppressor gene [56]. Snail has been shown to be one of the most important central regulators of epithelial cell adhesion during embryonic development [57]. Its transcriptional activity is positively correlated with cancer progression, mainly induced by metastasis, and various tumors have a poor prognosis [58]. Snail represses a variety of EMT-associated genes [59], and it also is a direct E-cadherin inhibitor [60]. Compound **4** led to a decreasing trend in the protein levels of Snail and Vimentin, and meanwhile an increase in E-cadherin protein levels in a dose-dependent manner, which suggests that compound **4** can inhibit the metastasis of TNBC cells, consistent with the findings from Zhang P et al. [61]. Wound-healing assays performed on both normal-growing MDA-MB-231 cells and drug-resistant MDA-MB-231 cells demonstrated a reduction in the cell migratory ability as the concentration of compound **4** increased, corroborating the results of the aforementioned experiments.

Considerable progress has been made in developing targeted cancer therapies over the past decades. We predicted the potential targets of compound **4** and identified MAP3K11 (also named MLK3) as a potential target. Molecular docking studies indicated that compound **4** would bind to the binding domain of the MLK3 target protein with strong binding activity, and it exhibited a binding energy of −8.0919 kcal/mol. MAP3K11 is a Ser/Thr protein kinase widely expressed in both normal and cancerous tissues, including the brain, lung, liver, heart, and skeletal muscle [62]. Studies have reported that MLK3 plays a significant role in various malignant tumors, including breast, cervical, colorectal, gastric, and prostate cancers [54]. In several cancer types, MLK3 signaling has been associated with promoting cell proliferation and driving cell migration [62]. MLK3 deficiency has been shown to reduce proliferation in vivo, while MLK3 inhibition decreases proliferation and colony formation [63]. In cervical cancer HeLa and SiHa cell lines, MLK3 knockdown resulted in reduced cell survival and increased apoptosis [64]. Additionally, MLK3 knockdown blocked JNK and ERK activation in human nerve sheath tumor cell lines, inhibiting cell proliferation. Similarly, the increase in cell proliferation observed in ovarian cancer cells could be reversed by knocking down MLK3 [65], which aligns with the corresponding decrease in cell proliferation and increase in apoptosis observed with increasing concentrations of compound **4** in our experiments. The inhibition or silencing of MLK3 can block the migration of highly migratory TNBC cells [66]. An earlier study by Mishra et al. found that cell migration depended on MLK3/MLKs activation, and pharmacological inhibition of MLK3/MLKs by CEP-1347 blocked breast cancer cell migration [67]. Furthermore, treatment with compound **4** significantly inhibited the migration of both MDA-MB-231 and drug-resistant MDA-MB-231 cells, supporting the role of MLK3 as a potential target of compound **4** in cell migration. MLK3 is crucial for microtubule stability during the G2/M transition, and the impairment of MLK3 results in the inability of cells to exit mitosis [68]. In HeLa cells, the inhibition of MLK3 activity led to G2/M arrest [69]. The inhibition of MLK3 activity with the MLK3 inhibitor CEP1347 resulted in a significant increase in cell populations in the G2/M phase in ovarian cancer cells, which could also lead to G2/M arrest in breast cancer cells [70]. Moreover, the results of the cellular thermal shift assay also proved that MLK3 is a potential target of compound **4**. These findings are consistent with the flow cytometry results observed in TNBC cells treated with compound **4**. Thus, it is evident that MKP3K11 is the most likely potential target of compound **4**.

## 4. Materials and Methods

### 4.1. Materials

#### 4.1.1. Cell Lines and Cell Culture

The breast cancer cell lines used in this study were cultured in Dulbecco’s Modified Eagle Medium (DMEM; Life Technologies, Carlsbad, CA, USA) supplemented with 10% fetal bovine serum (OriCell^®^ Therapeutics Holdings Ltd., Guangzhou, China) and 1% penicillin-streptomycin (NCM Biotech, Suzhou, China). All cells were maintained in a humidified environment with 5% CO_2_ at 37 °C. Breast cancer cells included MCF-7 (CTCC-001-0042), MCF-7/ADR (CTCC-0508-NY), MDA-MB-231 (CTCC-001-0019) [71], paclitaxel-resistant MDA-MB-231/TAX (CTCC-0539-NY) [72], normal mammary epithelial cells MCF-10A (CTCC-001-0045), THLE-2 (CTCC-004-0030) purchased from Zhejiang Meisen Cell Technology Co., Ltd (Zhejiang, China), and LX-2 (TCH-C391) purchased from Starfish Biology Co., Ltd. (Quanzhou, China). Adriamycin-resistant MDA-MB-231/ADR (LHY1610) [73] was purchased from Shanghai Lianmei Biological Engineering Co., Ltd. (Shanghai, China).

#### 4.1.2. Reagents and Antibodies

Compound **4** was dissolved in dimethyl sulfoxide (DMSO). Ultrasensitive ECL chemiluminescence detection kits were obtained from BSP Bioscience (San Diego, CA, USA). A cellular senescence β-galactosidase assay kit was purchased from Beyotime Biotechnology Institute (KeyGEN BioTECH, Nanjing, China). Antibodies for cleaved caspase-3 (ABP56699), caspase-7 (ABP50857), caspase-9 (ABP56744), ALDH1A1 (ABP52888), p16 (ABP52099), p21 (ABP57266), p27 (ABP53452), Vimentin (ABP52700), and CD133 (ABP52923) were acquired from Abbkine Scientific (Wuhan, China). Anti-rabbit IgG (D110058-0025) and CD44 (D161338-0025) were obtained from BBI Life Sciences Corporation (Shanghai, China). Snail (No. A1230) was purchased from ABclonal Technology (Wuhan, China), while β-tubulin (M30109XS) was purchased from Abmart (Shanghai, China). Anti-mouse IgG (No. RGAM001) and E-cadherin (NO.60335) were sourced from Proteintech Group (Wuhan, China). Phosphate-buffered saline (PBS) was obtained from VivaCell Biosciences (Shanghai, China). MAP3K11 (IPH1081) was purchased from baijia Biology Co., Ltd. (Tangshan, China).

### 4.2. Experimental Methods

#### 4.2.1. Compound Screening Sources

We screened four compounds isolated from the culture extracts of the sponge-associated *Streptomyces* sp. NBU3142, which were derived from the mesophotic zone in the ocean as reported previously [13,74]. Compounds **1**–**4** were isolated by our group. Compounds **1**,**2** have been reported in previous articles [13], while compounds **3**,**4** were newly isolated from *Streptomyces* sp. NBU3142 using the same isolation method, and these two compounds were identified by ^13^C nuclear magnetic resonance spectroscopy and ^1^H NMR to be consistent with the structures reported by previous authors [75] (Figure 1E–H).

Each compound was used at a concentration of 10 μM. The drugs were added after the cells had reached 70–80% confluency, and assays were conducted 48 h post-treatment.

#### 4.2.2. CCK8 Toxicity Assay

Cells were plated at a density of 7 × 10^3^ cells per well into 96-well microtiter plates (JET BIOFIL, Guangzhou, China) by adding 0.1 mL of medium and incubating for 24 h at 37 °C. Afterward, different concentrations of compound **4** were added (1, 3, and 10 μM) to 96-well plates and incubation occurred at 37 °C for 48 h. The structure of compound **4** was confirmed by mass spectrometry. Cell viability was assessed using a colorimetric assay, wherein 10 μL of CCK8 (NCM Biotech, Suzhou, China) solution was added to each well, and the plates were incubated at 37 °C with 5% CO_2_ for 1 to 4 h. Absorbance values were measured at 450 nm using a microplate reader (Molecular Devices, San Jose, CA, USA). The effect of each treatment is expressed as the percentage of viable cells relative to the untreated control. All experiments were conducted in triplicate.

#### 4.2.3. Apoptosis Analysis

MDA-MB-231 and MDA-MB-231/Tax cells were inoculated into 6-well plates (Corning, New York, NY, USA) at 8 × 10^5^ cells per well. Once the cells reached 80% confluence, the medium was aspirated, and the cells were washed once with PBS. The drug was diluted in 1% DMEM, and the cells were incubated with varying concentrations of compound **4** (1, 3, and 10 μM) for 24 h at 37 °C [76]. Apoptosis was detected using PI and Hoechst33342 (Bioworld, Nanjing, China) following a modified protocol by Young et al. [77]. Briefly, samples were fixed with paraformaldehyde, and 10 μL of Hoechst33342 was added to 1 mL of medium and incubated for 5–15 min at 37 °C. After washing with PBS, 1.0 mL of Buffer A working solution was added, followed by 5 μL of PI staining solution [78]. The mixture was incubated for 5–15 min at room temperature. After washing the samples twice with PBS, they were mounted in a sealer and examined with a fluorescence microscope (Nikon, Tokyo, Japan). Monochrome fluorescence images were merged and quantitatively analyzed using ImageJ software 1.8.0 (National Institutes of Health, Bethesda, MD, USA).

#### 4.2.4. Cellular Senescence Assay

The cellular senescence assay was conducted with slight modifications from previously established methods [79]. Specifically, MDA-MB-231 and MDA-MB-231/Tax cells were inoculated in triplicate into 6-well plates at a density of 8 × 10^5^ cells per well. Once the cells reached approximately 80% confluence, compound **4** was diluted in 1% DMEM and applied in a gradient (1, 3, and 10 μM) for a duration of 24 h. For the senescence assay, the following protocol was employed: First, the cell culture medium was aspirated, and the cells were washed once with PBS. Next, 1 mL of β-galactosidase staining fixative was added to each well, and the cells were fixed at room temperature for 15 min. The fixative was then aspirated, and the cells were washed three times with PBS, with each wash lasting 3 min. After removing the PBS, 1 mL of staining working solution was added to each well. The staining working solution was prepared according to the following: β-galactosidase staining solution A (10 μL), β-galactosidase staining solution B (10 μL), β-galactosidase staining solution C (930 μL), and X-Gal solution (50 μL). The plates were incubated overnight at 37 °C, and to prevent evaporation, the 6-well plates were sealed with cling film. Finally, the stained cells were examined under a microscope (Nikon, Tokyo, Japan) to observe and count blue-stained cells indicative of senescence. This detailed methodology outlines the process for assessing cellular senescence using β-galactosidase activity, which serves as a biomarker for aging cells.

#### 4.2.5. Cancer Cell Stemness Assay

Both the MDA-MB-231 and MDA-MB-231/Tax cells were treated with compound **4**, washed once with PBS, and then digested. A total of 1 × 10^6^ cells were collected and divided into several groups: a blank group (containing only the cell suspension), a CD44 single-stained group (using antibodies from Multi Sciences Biotech, Hangzhou, China), a CD24 single-stained group (using antibodies from Thermo Fisher Scientific, Waltham, MA, USA), and an experimental group (consisting of a cell suspension with FITC-labeled mouse anti-human CD44 and APS-labeled mouse anti-human CD24 antibodies).

For each group, 2 μL of the corresponding antibody was added, and the samples were incubated for 30 min at room temperature in the dark. Following incubation, the cells were washed with PBS and centrifuged at 500 g for 5 min to remove excess antibodies. The cell populations were then sorted using a Beckman Coulter flow cytometer (Pasadena, CA, USA), and the data were analyzed using FlowJo_V10 software (Tree Star Inc., Ashland, OR, USA).

#### 4.2.6. Cell Cycle Analysis

The effects of compound **4** on DNA content and cell cycle distribution in the MDA-MB-231 and MDA-MB-/Tax cell lines was assessed through propidium iodide (PI) staining, following the protocol previously described [80]. Briefly, cells were plated at a density of 8 × 10^5^ cells/mL in 6-well plates. After 24 h, both floating and adherent cells were collected, and a cell pellet containing 1 × 10^6^ cells was washed with PBS and fixed in pre-cooled 70% ethanol overnight. The cells were then precipitated, washed with PBS again, and stained with a solution of 50 μg/mL PI (Sigma) combined with 100 μg/mL RNase (Sigma, St. Louis, MO, USA). The cells were gently resuspended and incubated in the dark at 37 °C for 30 min. The cell cycle distribution was examined using a Beckman Coulter flow cytometer and analyzed with FlowJo_V10 software.

#### 4.2.7. Wound-Healing Assay

MDA-MB-231 and MDA-MB-231/Tax cells (8 × 10^5^ cells per well) were inoculated in 6-well plates. After 24 h, the cells were allowed to adhere to the wells, washed in PBS, and floating cells were isolated with PBS after delineating a uniformly sized band in each well. The cells were then treated with compound **4** and dissolved in DMSO, at concentration gradients of 0, 1, 3, and 10 μM. At 0 h and 24 h post-drug administration, cell migration was observed microscopically across the wound area. The migration rate was calculated using ImageJ software 1.8.0.

#### 4.2.8. Western Blotting

Following the drug treatment of cells in the 6-well plates, both dead and adherent cells were collected in the original medium and lysed on ice for 30 min using RIPA l buffer containing 1% protease inhibitor. After sufficient lysis, the samples were centrifuged at 13,000 rpm for 15 min [81]. The supernatant was collected, and protein concentration was determined using a bicinchoninic acid (BCA) protein assay kit (Thermo Scientific, Waltham, IL, USA) by mixing an equal volume of protein with 1× protein loading buffer and heating at 100 °C for 15 min. Proteins from each sample were separated via 10–15% SDS-PAGE and incubated at room temperature in TBST containing 5% skimmed milk for 2 h. The membranes were washed with TBST for 10 min, repeated five times, and subsequently transferred to polyvinylidene fluoride (PVDF) membranes. The membranes were incubated overnight at 4 °C with a primary antibody (1:1000), followed by washing with TBST. A goat anti-rabbit HRP secondary antibody (1:2000) was then incubated for 1.5 h at room temperature and washed again with TBST. ECL chemiluminescent substrate was utilized to visualize the protein bands, which were detected using the Tanon 5200 Luminescent Imaging System. Each Western blotting analysis was performed using ImageJ software 1.8.0, and the images of the bands were quantified and normalized.

#### 4.2.9. Molecular Docking and Virtual Screening

The initial model of compound **4** with the target was deduced from the X-ray crystal structure (PDB: 3DTC) using the molecular transformation procedure implemented in the ICM-pro3.6-1d program (Molsoft, San Diego, CA, USA). The molecular transformation procedure and high-throughput molecular docking were carried out as previously reported [82,83]. The 2D structure of the small-molecule ligand was obtained from the PubChem database (http://pubchem.ncbi.nlm.nih.gov/, accessed on 2 August 2024), and the 2D structure was imported into the ChemOffice 20.0 software to produce its 3D structure, which was saved as a mol2 file [84]. Then, the RCSB PDB database (http://www.rcsb.org/, accessed on 2 August 2024) was applied to screen the crystal structure of the protein target with high resolution as the molecular pair acceptor, and the PyMOL 2.6.0 software was used to dehydrate and dephosphorylate the protein and save it as a PDB file. Molecular Operating Environment 2019 software was used to minimize the energy of the compounds, pre-process the target proteins, and find the active pockets. Finally, MOE 2019 was run for molecular docking with the number of operations set to 50. The binding activity of both was evaluated based on the magnitude of binding energy, and the results were visualized by PyMOL 2.6.0 and Discovery Studio 2019 software. Compound **4** potential target prediction was performed using target prediction websites based on three different algorithms. In Target Hunter (http://www.cbligand.org/TargetHunter, accessed on 2 August 2024), Chem Mapper (https://www.lilab-ecust.cn/chemmapper/login.html, accessed on 2 August 2024), Super Target (https://prediction.charite.de/subpages/target_prediction.php, accessed on 2 August 2024), the structural formula of compound **4** was entered, a series of possible targets were listed by different algorithms, and the targets listed by the three algorithms were used to identify concurrent genes by intersection. The identified targets were analyzed in the breast cancer database bc-GenExMiner v5.1 (https://bcgenex.ico.unicancer.fr/BC-GEM/GEM-requete.php, accessed on 2 August 2024), and the RNA sequences of the targets were analyzed by selecting “Expression Analysis” and selecting TNBC (IHC) subtypes vs. non-TNBC (IHC) to study the RNA expression levels of the targets in TNBCs and non-TNBCs. 

#### 4.2.10. Cellular Thermal Shift Assay

Compound **4** was added to the cells, and after a period of time, the cell suspension was collected and the samples were heated in a PCR amplifier for 5 min to 45, 50, 55, 60, 65, or 70 °C [21]. The heat-treated samples were allowed to stand at room temperature for 3 min, and the heated samples were lysed by repeated freeze-thawing in liquid nitrogen and in a water bath at 37 °C. The supernatant was removed by centrifugation at 4 °C for 15 min, 14,000× *g*. Protein loading was added and boiled for 15 min. The supernatant was removed by centrifugation at 4 °C, 14,000× *g* for 15 min, and the supernatant was added to the protein loading and boiled for 15 min [85].

#### 4.2.11. Statistical Analyses

All statistical analyses were conducted using GraphPad Prism 5 (GraphPad, San Diego, CA, USA). *T*-tests were employed to assess the statistical significance between two groups. Comparisons involving multiple groups were analyzed using ANOVA, while two-way ANOVA was used for comparisons between different groups. Error bars, unless stated otherwise, represent the standard error of the mean (SEM) from three experiments. A *p*-value of less than 0.05 was considered statistically significant.

## 5. Conclusions

**Figure 8:**
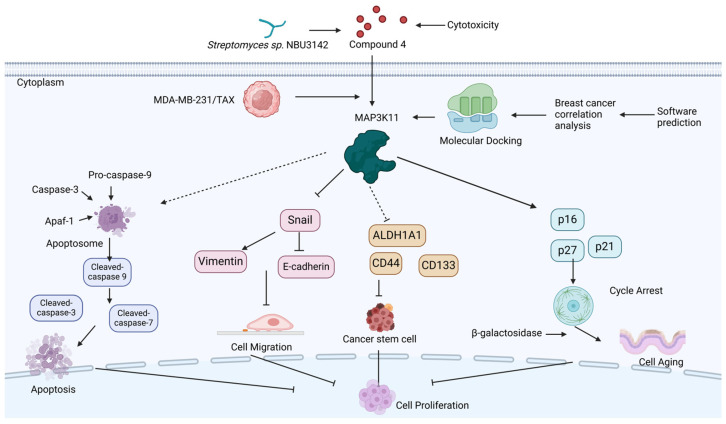
The proposed anticancer action mechanisms of compound **4** against TNBC cells. This figure was produced by BioRender (ID: VC277ZD2U8).

In summary, compound **4** exhibited the most pronounced inhibitory effect on TNBC cells, including chemoresistant cell lines. Mechanistically, compound **4** exerts its anticancer activity via targeting MKP3K11 and thus promoting apoptosis, inhibiting cell proliferation by reducing the ratio of breast cancer stem cells and inducing cellular senescence by facilitating cell cycle arrest in the G2/M phase and decreasing migratory activity (Figure 8). Collectively, these results underscore the promising potential of staurosporine analog **4** as a novel drug candidate for TNBC therapy.

## Data Availability

All raw data are readily available upon request.
